# Time-course effects of cognitively engaging physical activity on executive function and self-control in younger school-aged children: a randomized controlled trial

**DOI:** 10.3389/fpsyg.2025.1628814

**Published:** 2025-08-29

**Authors:** Yifan Xu, Kai Qi, Marcin Białas, Qi Xu, Xiaodan Guo, Aiguo Chen

**Affiliations:** ^1^Gdansk University of Physical Education and Sport, Gdańsk, Poland; ^2^Nanjing Sport Institute, Nanjing, China

**Keywords:** cognitive-motor intervention, executive function, self-control, younger schoolaged children, time-course effects

## Abstract

**Objective:**

This study aimed to investigate the time-course effects of cognitively engaging physical activity (CEPA) on executive function (EF) and self-control in younger school-aged children (ages 8–10 years) exploring the differential impacts and temporal dynamics of the intervention.

**Methods:**

Using a cluster randomized controlled trial design, 203 younger school-aged children (age = 8.9 ± 0.67, male 54.7%, female 45.3%) were randomly allocated into either an experimental group receiving CEPA or a control group receiving the traditional physical education curriculum. The intervention lasted 10 weeks, occurring three times per week, 45 min per session, structured according to a “graded cognitive load” principle, progressively increasing cognitive complexity and challenges. Assessments of EF (inhibitory control, working memory, cognitive flexibility) and self-control (social interaction, emotional experience, learning behaviors, daily habits) were conducted at baseline, immediately post-intervention, and at a three-month follow-up. Linear mixed-effects models were used to account for the clustered study design.

**Results:**

Linear mixed-effects models revealed significant time-by-group interaction effects for executive function components: inhibitory control (*p* = 0.020, ICC = 0.057) and working memory (*p* = 0.002, ICC = 0.054), with cognitive flexibility showing a trend toward significance (*p* = 0.077, ICC = 0.000). A significant interaction effect was also observed for the total self-control score (*p* < 0.001, ICC = 0.040). The experimental group demonstrated significant improvements in executive function, with working memory showing substantial immediate gains, and inhibitory control exhibiting strong retention. Regarding self-control dimensions, the most pronounced improvements occurred in social interaction (*p* < 0.001, ICC = 0.000) and learning behavior (*p* < 0.001, ICC = 0.072). Overall, intervention effects displayed a nonlinear time-course, characterized by rapid improvements during the intervention phase, slight attenuation at follow-up but remaining significantly above baseline levels, whereas no significant changes were observed in the Traditional Physical Education Curriculum control group.

**Conclusion:**

This study systematically confirms the immediate and lasting effectiveness of CEPA on younger school-aged children’s executive function and self-control, highlighting the differential effects across cognitive and behavioral domains and their nonlinear temporal characteristics. These findings underscore the value of integrating cognitive engagement elements into the school-based physical education curriculum, offering robust empirical support for educational practices and policy decisions aimed at comprehensive cognitive and behavioral development among children.

## 1 Introduction

Childhood executive function (EF) development significantly influences cognitive growth, academic achievement, and social–emotional capabilities (Korzeniowski et al., 2021). EF is a higher-order cognitive function, enables children to engage in goal-oriented behavior, maintain mental flexibility, and achieve effective regulation of behaviors and emotions (Diamond, 2013). Diamond’s classic definition identifies three core components of executive function: inhibitory control (ability to control behavior and attention), working memory (ability to maintain and manipulate information), and cognitive flexibility (ability to switch flexibly between tasks or thinking patterns) (Karbach and Kray, 2020). Research indicates that the younger school-aged period, especially between 8 to 10 years, represents a critical window for executive function (EF) development (Hughes and Ensor, 2011; Pureza et al., 2013; Vuontela et al., 2012). The prefrontal cortex experiences notable neuroplasticity during this period, forming the biological foundation for rapid EF development (Best et al., 2009). Numerous empirical studies reveal substantial positive correlations between strong EF development and children’s reading comprehension, mathematical achievement, peer relationship quality, and emotional regulation ability (Blair and Razza, 2007; Bull and Lee, 2014). Current research, however, shows approximately 15–20% of school-aged children, including younger school-aged children, experience delayed EF development, mainly characterized by attention deficits, impulse control difficulties, and impaired task-switching abilities (Chung et al., 2017). These EF deficits directly impact children’s academic performance and may lead to long-term social adaptation issues and emotional difficulties, with severe cases potentially elevating the risk of future mental health problems (Diamond and Lee, 2011; Shroff et al., 2023; Zelazo and Doebel, 2015). Promoting effective EF development in younger school-aged children has consequently become a core focus within education and public health fields.

In addition to EF, self-control, as an important ability for children to regulate emotions, behaviors and impulses in real situations, also has a profound impact on children’s academic performance and social adaptation (Nigg, 2017). Theoretically, self-control represents EF in daily life situations as a form of “hot executive function,” sharing a common neural basis with EF, particularly within the prefrontal cortex (Zelazo and Carlson, 2012). Previous studies indicate strong EF often co-occurs with high self-control abilities, and both collectively predict children’s academic achievement and social adaptation levels (Gestsdóttir et al., 2023). Although research on promoting children’s EF development has gradually increased, exploration of how to effectively enhance self-control remains limited.

Cognitive Exercise Physical Activity (CEPA), a novel intervention combining physical activity and cognitive training, has shown distinct advantages in promoting children’s cognitive development in recent years. CEPA promotes cognitive engagement during exercise through cognitive elements like decision-making, strategy adjustment, and attention control (Mao et al., 2024; Mavilidi et al., 2022). Compared with the traditional physical education curriculum, which emphasizes repetitive movement practice and basic skill training, CEPA simultaneously activates brain motor regions and cognitive control networks, creating synergistic effects that enhance executive function (Tomporowski et al., 2015a; Tomporowski et al., 2015b). The theoretical mechanisms behind CEPA enhancing EF primarily include two aspects: neuroplasticity theory, which suggests exercise strengthens prefrontal cortex connections via increased Brain-Derived Neurotrophic Factor (BDNF) secretion, thereby providing a biological basis for cognitive development (Hillman et al., 2008), and cognitive engagement hypothesis, which posits that combined physical and cognitive activities more effectively activate EF-related brain regions (Best, 2010), thus improving EF performance.

Multiple empirical studies have confirmed the beneficial effects of CEPA on children’s EF. For example, Pesce’s 8-week group game intervention, incorporating strategic thinking elements, notably improved working memory and cognitive flexibility among 8-10-year-old children (Pesce et al., 2019). Schmidt’s study indicated that “exercise + thinking” curricula, integrating cognitive components, resulted in greater improvements in inhibitory control and attention-switching abilities among school-aged children compared with Traditional Physical Education Curriculum (Schmidt et al., 2015). Moreau found that team sports involving decision-making and tactical thinking yielded stronger EF enhancement effects compared to purely aerobic training (Moreau and Conway, 2014). Despite preliminary validation of cognitive exercise interventions’ positive effects, current research has three primary limitations: first, most studies adopt single-measurement designs, failing to capture temporal characteristics of intervention effects, such as developmental trajectories and maintenance durations, second, existing intervention programs often combine singular cognitive tasks with exercise but lack systematic integration of multiple EF components. To address these limitations, this study innovatively employs a “gradient cognitive load” intervention design, progressively increasing cognitive task complexity over a 10-week intervention period. Compared with traditional fixed-difficulty interventions, this progressive approach better aligns with the “moderate challenge” principle of brain plasticity, potentially yielding more sustained intervention effects (Pesce and Ben-Soussan, 2023).

Furthermore, limited studies indicate that motor interventions promoting EF development may also enhance self-control in younger school-aged children (Koepp and Gershoff, 2022). For instance, motor activities involving emotion suppression, impulse control, or social decision-making require children to continuously adjust behaviors to adapt to environmental changes, potentially facilitating self-control development. However, systematic investigation of CEPA on self-control remains lacking. Therefore, further exploration of cognitive motor intervention temporal effects on these two variables will clarify the intervention mechanism and provide scientific evidence for designing more effective interventions to enhance younger school-aged children’s cognitive and behavioral development.

Based on the above research gap, this study aims to investigate the temporal effects of a 10-week CEPA on executive function and self-control in younger school-aged children. This study establishes three measurement points (pre-intervention, immediately post-intervention, and 3-months post-intervention) to comprehensively assess both immediate and sustained intervention effects. Based on the theoretical framework and prior research, this study proposes the following hypotheses:

1. CEPA will enhance the EF of younger school-aged children, and this enhancement effect can last until 3 months after the end of the intervention.
2. Among the three core EF components, inhibitory control will demonstrate the greatest and most sustained improvement, followed by working memory, while cognitive flexibility will exhibit relatively smaller improvements.
3. Improvements in EF will follow a nonlinear trajectory, characterized by faster improvement during the intervention period (weeks 0–10) than during the maintenance phase (weeks 10 to 3 months post).
4. CEPA will enhance the self-control of younger school-aged children, and this enhancement effect can last until 3 months after the end of the intervention.

This study’s innovation is reflected in several aspects: First, it employs a longitudinal experimental design to systematically examine both the immediate and long-term maintenance effects of CEPA for the first time. Second, this study incorporates self-control, a key variable of “hot EF,” into the research framework to explore the comprehensive impact of CEPA on children’s EF and their performance in real life situations transformation; third, it introduces the innovative “gradient cognitive load” intervention model. Theoretically, this study will deepen understanding of the interaction between cognitive function and behavioral development, offering a new perspective for future EF intervention design. Practically, the findings will provide an evidence-based foundation for reforming school physical education curricula and offer scientific guidance to policymakers, facilitating the integration of CEPA into regular education systems.

## 2 Materials and methods

### 2.1 Study design

This study adhered to CONSORT guidelines for randomized controlled trials and employed a cluster randomized controlled design comprising two groups (experimental and control) across three assessment points: pre-test, post-test, and a follow-up 3 months after post-test completion (Schulz et al., 2010). Four classes were randomly selected from registered participants via a computer-generated random number table and assigned to either the experimental group (CEPA) or the control group (Traditional Physical Education Curriculum). Outcome measures included EF (inhibitory control, working memory, cognitive flexibility) and self-control (Lifestyle Habits, Social Interaction, Emotional Experience, Learning Behavior).

### 2.2 Participants

Using version 3.1.9.7 of G*Power, the effect size *f* = 0.25, significance level *α* = 0.05, power = 0.80, the number of inter-group measurements = 2, and the number of repeated measurements = 3 were set, and the required total sample size *N* = 42 was calculated.

Inclusion criteria were as follows: (1) the ability to safely participate in regular physical education classes, confirmed by school health records and teacher reports; (2) no prior exposure to cognitively engaging physical activity (CEPA) or structured psychological interventions; and (3) written informed consent provided by legal guardians and child assent was obtained following age-appropriate explanations. Exclusion criteria included: (1) diagnosed psychological, neurological, or developmental disorders (e.g., ADHD, ASD, depression); (2) medical contraindications or physical conditions (e.g., recent injury, chronic illness) affecting safe physical activity; and (3) participation in other structured physical or psychological programs within the past 3 months. These criteria were intended to control for confounding variables that could bias the interpretation of intervention effects.

During the intervention, all participants were explicitly instructed not to engage in any additional extracurricular physical or psychological training. Compliance was monitored weekly through communication with teachers and parents to ensure intervention exclusivity and to control for external confounds.

**Figure 1:**
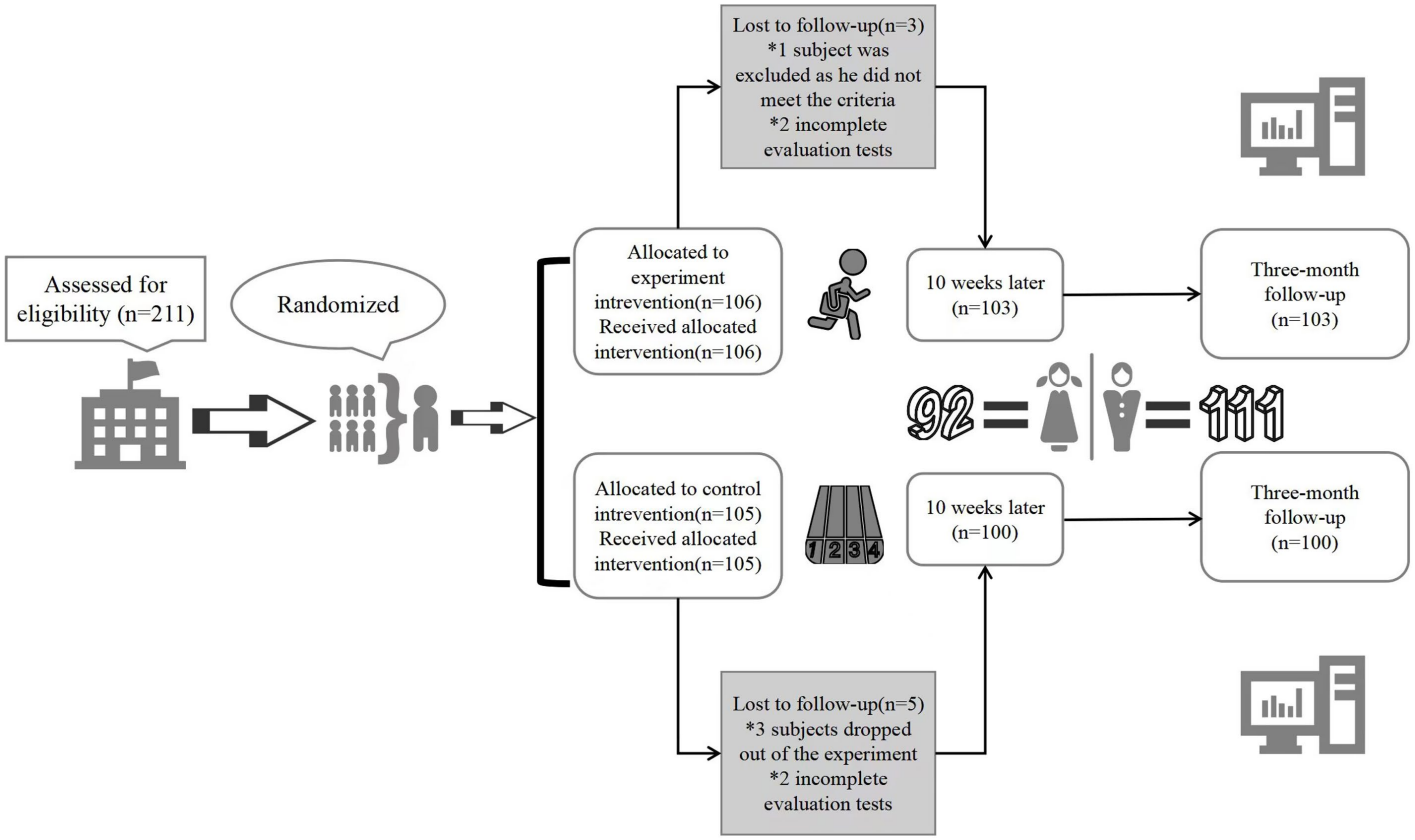
Flowchart of participant selection.

A total of 211 participants were initially enrolled. Among these, one was excluded due to not meeting the criteria, three withdrew during the study, and four failed to complete assessments. Ultimately, 203 participants (92 females, 111 males; ages 8–10 years, mean age 8.9 ± 0.67 years) completed the full experimental protocol, including 103 in the experimental group and 100 in the control group. Detailed procedures are illustrated in Figure 1.

Written informed consent was obtained from participants’ legal guardians prior to enrollment. Additionally, children received a clear and age-appropriate explanation of the study procedures and were encouraged to express their willingness to participate and ask questions. The study was conducted in accordance with the ethical standards outlined in the Declaration of Helsinki and was approved by the Ethics Review Committee of Yangzhou University School of Medicine (Approval No. YXYLL-2023-129). This study was registered in the Chinese Clinical Trial Registry (ChiCTR) with the registration number ChiCTR2500101607.^1^

### 2.3 Materials

#### 2.3.1 Executive function

The EF assessment employed a computerized neuropsychological system developed by Professor Chen specifically for Chinese children (Chen et al., 2011), ensuring cultural relevance and ecological validity for the target population. This system has been widely used in previous studies (Sun et al., 2024; Zhu et al., 2023), is appropriate for primary school students, and allows efficient group administration. Participants completed a guided practice session with the chief examiner before beginning the formal assessment. All participants were instructed to respond as quickly as possible while maintaining accuracy.

**Figure 2:**
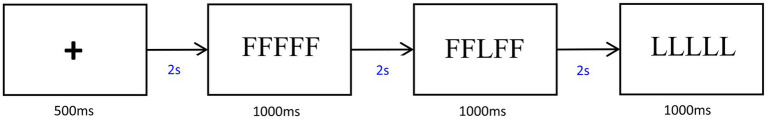
Flanker task.

The Flanker task began with a fixation cross (“+”) displayed for 500 milliseconds. Next, a letter array appeared centrally on the screen under either consistent (“FFFFF,” “LLLLL”) or inconsistent (“LLFLL,” “FFLFF”) conditions. Each was displayed for 1,000 milliseconds, with a 2-s interval between stimuli. Both conditions were presented randomly and in equal proportion, as illustrated in Figure 2. Participants were required to quickly and accurately identify the central letter: press the “F” key for “F” or the “L” key for “L” using the index finger. After 12 practice trials, participants completed 48 formal trials. Inhibitory control was calculated by subtracting the reaction time in consistent trials from that in inconsistent trials. A smaller difference indicates better inhibitory control.

**Figure 3:**
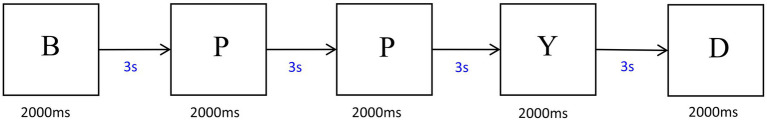
1-back task.

The 1-back task used five English letters (“B,” “D,” “L,” “Y,” “P”) as stimuli. After the task began, each letter was individually presented at the center of the screen. Each stimulus was displayed for 2 s, followed by a 3-s interstimulus interval, as shown in Figure 3. Participants were asked to determine whether the current letter matched the one presented immediately before. If the letters matched, the “F” key was pressed; otherwise, the “J” key was used. After 12 practice trials, participants completed 25 formal trials. Working memory performance was measured by the average reaction time. Shorter reaction times indicated better working memory capacity.

**Figure 4:**
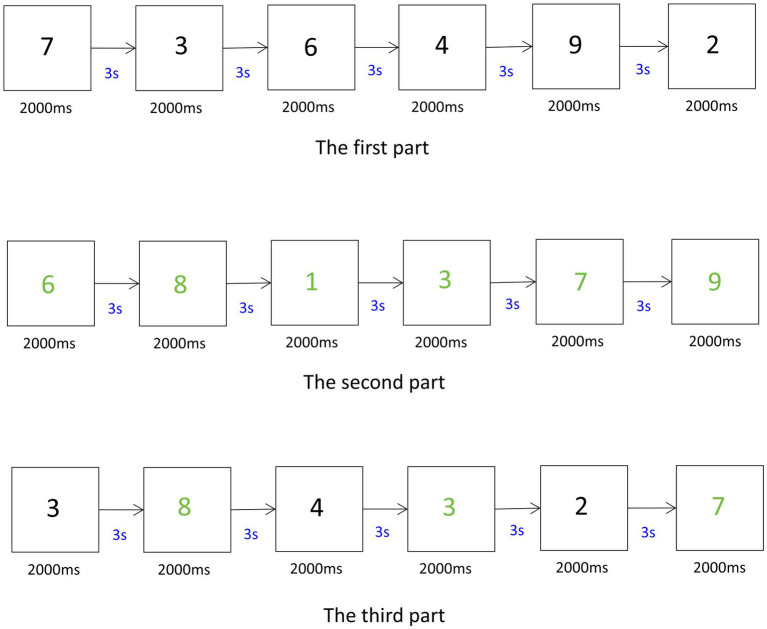
More-odd shifting task.

The More-Odd Shifting Task consisted of three parts. Part One (Numerical Judgment): Participants judged the magnitude of black numbers (1–9, excluding 5) presented on the screen center. The “D” key was pressed for numbers greater than 5, and the “F” key for numbers less than 5. Part Two (Parity Determination): Participants determined the parity of green numbers (1–9). Odd numbers required a “J” key response, even numbers required the “K” key. Part Three (Mixed Judgment): Tasks varied by color—black numbers required magnitude judgments, green numbers required parity judgments. Response keys remained the same as in the previous sections. Participants were instructed to respond as quickly as possible while maintaining accuracy. Each stimulus was displayed for 2000 milliseconds, with a 3-s interval between trials, as shown in Figure 4. Parts One and Two included 8 practice and 16 formal trials each. Part Three included 16 practice and 32 formal trials. Cognitive flexibility was calculated by subtracting the average reaction time of Parts One and Two from that of Part Three. Smaller differences indicated better cognitive flexibility.

#### 2.3.2 Self-control

Self-control was assessed using the “Student Self-Report Scale for Children’s Self-control,” developed by Chinese scholar Liu Jinhua (Liu et al., 1998). The Chinese version has been extensively validated and applied in studies involving Chinese children, ensuring its validity and cultural relevance for this study. The scale consists of 38 items, measuring four dimensions: learning behavior, behavioral habits, social interaction, and self-control quality. It uses a Likert 5-point scale (1 to 5 points), reverse scoring for certain items, and summing the scores of all items to obtain a total score. Higher scores indicate stronger self-control ability. The Cronbach’s *α* coefficient for the scale in this study was 0.847.

### 2.4 Procedure

All measurements were conducted during the standard two-week teaching period. To ensure the consistency and reliability of the results, the study strictly controlled three factors: environment, measurement personnel, and pre-measurement preparation. EF measurements were conducted uniformly in a quiet, undisturbed computer room, while self-control scales were completed under the guidance of trained researchers in a quiet classroom to ensure independent completion. The measurement team comprised four professionals with master’s degrees. All team members underwent systematic training prior to data collection, covering the operation of measurement tools, standardized instructions, and data recording methods. Prior to the measurement, all participants were given standardized test instructions and exercises to ensure understanding of the measurement requirements.

### 2.5 Intervention

**Figure 5:**
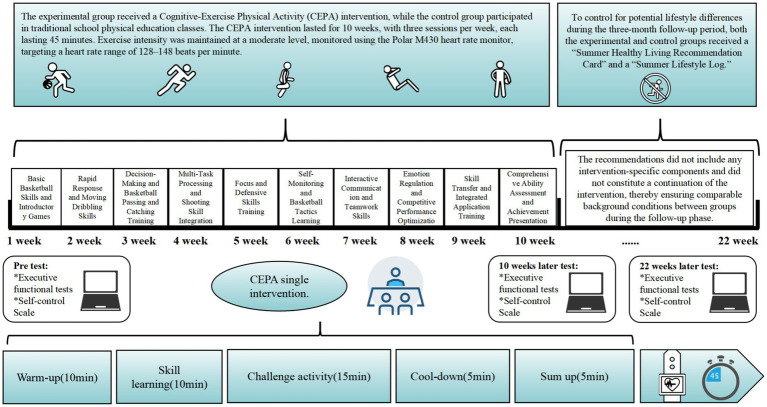
Class arrangement.

Based on empirical studies and meta-analytic findings indicating that at least 8 weeks of intervention are required to produce significant effects (de Greeff et al., 2018; Xu et al., 2023), this study designed a 10-week intervention cycle and innovatively set up a three-month follow-up measurement after the intervention to comprehensively evaluate the persistence of the intervention effect. Both the experimental and control groups received interventions that occurred three times a week for 45 min per session, both maintaining moderate intensity (heart rate monitored using Polar M430, with a target range of 128–148 beats per minute). The experimental group focused on CEPA, integrating cognitive challenges into sports, including team games requiring concentration and rapid decision-making, coordination exercises emphasizing memory-movement integration, and interactive sports requiring continuous task or movement pattern adjustments. This integration of cognitive tasks aimed to enhance students’ cognitive abilities and psychological adjustment, while also promoting overall physical fitness. The class and test process of the experimental group is shown in Figure 5. The control group participated in traditional physical education courses, focusing on basic physical training, regular sports games, and motor skills practice. The complete 10-week course plan is shown in Appendix 1.

To minimize the potential influence of the instructor, both groups were taught by the same teacher. Instructors were regular physical education teachers who underwent strict training to ensure that the experimental group followed the intervention plan, while the control group received standard instruction. Researchers supervised the entire experimental process to ensure the intervention plan was implemented. After the 10-week intervention, a three-month summer break followed. To control differences in summer lifestyles, both groups received a “Summer Healthy Life Suggestion Card” and a “Summer Life Record Form” to guide students in maintaining basic physical activity. The suggestions did not include specific intervention components or constitute continuous intervention, ensuring consistency between the groups during the follow-up phase.

### 2.6 Statistical analysis

Data were imported into SPSS 29.0 for processing. Descriptive statistics are reported as mean and standard deviation. The Kolmogorov–Smirnov test and *P–P* plot were used to assess data normality. The results indicated that the data approximately followed a normal distribution. The Levene test was applied to assess data homogeneity. Independent t-tests and Cohen’s d effect size tests compared baseline levels between groups.

Given the cluster randomized design, linear mixed-effects models were used to analyze the data to account for the clustered nature of the study design (students nested within classes). Time, group, and their interaction were treated as fixed effects, while class was included as a random effect to control for intraclass correlation. The models were fitted using restricted maximum likelihood (REML) estimation. Intraclass correlation coefficients (*ICC*) were calculated to quantify the degree of clustering within classes. Effect sizes for within-group changes were calculated using Cohen’s *d* (Cohen, 1992), with thresholds defined as follows: >0.2 (small), >0.5 (medium), and >0.8 (large). Post-hoc comparisons were conducted using the Bonferroni correction. All statistical analyses were conducted using SPSS 29.0, with the predetermined significance level set at *p* < 0.05.

## 3 Result

### 3.1 Baseline assessments

A total of 203 participants who completed all tests were included in this study: 103 in the experimental group and 100 in the control group. No significant differences were found between the experimental and control groups in gender, age, EF (inhibitory control, working memory, cognitive flexibility), and self-control (Lifestyle Habits, Social Interaction, Emotional Experience, Learning Behavior), indicating good homogeneity before the intervention. Basic information for both groups is shown in Table 1.

**Table 1:** Characteristics of the study participants.

Measure	Experimental group (*n* = 103)	Control group (*n* = 100)	*t*	*p*
Male	57 (55%)	54 (54%)	0.191	0.849
Female	46 (45%)	46 (46%)	0.191	0.849
Age (years)	8.90 ± 0.679	8.89 ± 0.680	0.135	0.892
Inhibitory control	40.26 ± 10.47	39.95 ± 10.99	0.204	0.838
Working memory	1048.62 ± 228.20	1103.15 ± 233.64	−1.682	0.094
Cognitive flexibility	551.18 ± 149.76	547.26 ± 148.53	0.187	0.852
Lifestyle habits	22.10 ± 4.20	22.04 ± 4.12	0.098	0.922
Social interaction	18.07 ± 3.62	17.99 ± 3.69	0.152	0.879
Emotional experience	9.01 ± 3.33	8.53 ± 3.35	1.024	0.307
Learning behavior	59.97 ± 9.94	59.94 ± 10.06	0.022	0.982
Self control	109.15 ± 11.65	108.61 ± 12.04	0.322	0.748

Data were expressed as mean ± standard deviation (M ± SD); *p-*value indicates the significance of the difference between groups; and * indicates *p* < 0.05.

Given the clustered nature of the study design, intraclass correlation coefficients (ICC) were calculated to assess the degree of clustering within classes. ICC values ranged from 0.000 (cognitive flexibility and social interaction) to 0.072 (learning behavior), indicating minimal to small clustering effects within classes (Koo and Li, 2016). The presence of clustering justified the use of linear mixed-effects models to account for the non-independence of observations within classes.

### 3.2 The intervention effect on EF

#### 3.2.1 Inhibitory control

**Figure 6:**
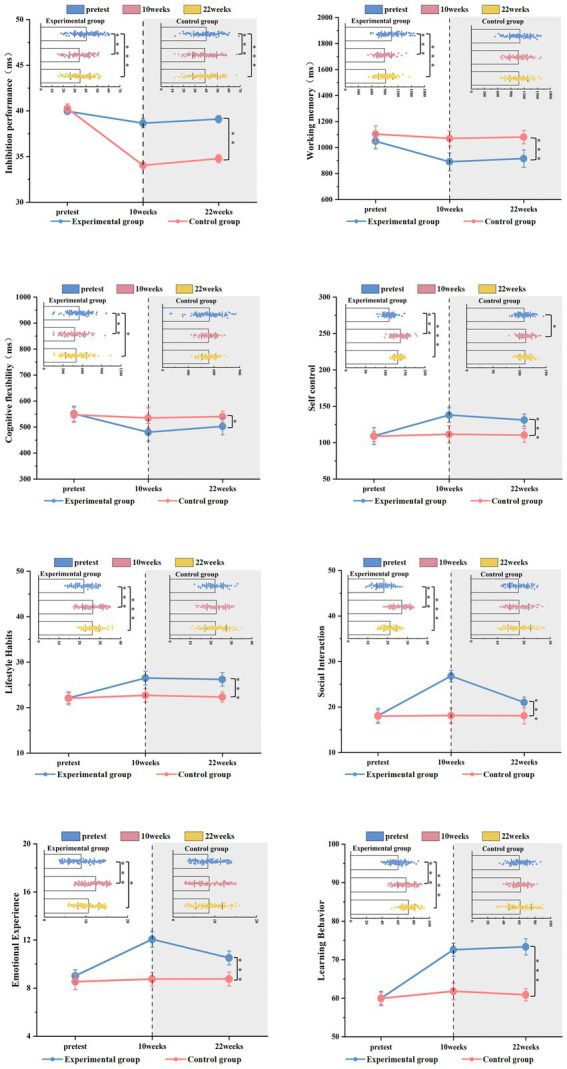
Within-group changes and between-group comparisons.

Linear mixed-effects model analysis revealed a significant time × group interaction for inhibitory control [*F*(2, 598.94) = 3.938, *p* = 0.020, ICC = 0.057], indicating differential change patterns between groups across time points (see Figure 6).

Further simple effect analysis showed significant within-group changes for the experimental group: performance significantly improved from baseline to post-test (*t* = 6.233, *p* < 0.001) and from baseline to follow-up (*t* = 5.493, *p* < 0.001), corresponding to a 15.47% reduction in response time at post-test (*d* = −0.66) and a sustained 13.64% improvement at follow-up (*d* = −0.58). The control group showed minimal changes across all time points.

Between-group comparisons revealed no significant differences at baseline. However, the experimental group demonstrated significantly better inhibitory control performance than the control group at both post-test and follow-up, confirming the sustained effectiveness of CEPA intervention. Table shows the descriptive statistics and effect sizes for both groups across all time points.

#### 3.2.2 Working memory

Linear mixed-effects model analysis revealed a significant time × group interaction for working memory [*F*(2, 598.8) = 6.091, *p* = 0.002, ICC = 0.054], indicating differential change patterns between groups across time points (see Figure 6).

Further simple effect analysis showed significant within-group changes for the experimental group: performance significantly improved from baseline to post-test (*t* = 158.621, *p* < 0.001) and from baseline to follow-up (*t* = 133.622, *p* < 0.001), corresponding to a 15.13% reduction in reaction time at post-test (*d* = −0.80) and a sustained 12.74% improvement at follow-up (*d* = −0.66). The control group showed minimal changes across all time points.

Between-group comparisons revealed no significant differences at baseline. However, the experimental group demonstrated significantly better working memory performance than the control group at both post-test and follow-up, confirming the sustained effectiveness of CEPA intervention. Table 2 shows the descriptive statistics and effect sizes for both groups across all time points.

**Table 2:** Descriptive statistical analysis and mixed-effects model results for executive function measures.

Measure	Group	Pre-test	Post-test	Follow-up	Pre-post change	Pre-follow-up change	Mixed-effects model results
Inhibitory control	Experimental	40.26 ± 10.47	34.03 ± 8.63	34.77 ± 8.81	−15.47%; *d* = −0.66	−13.64%; *d* = −0.58	*F*(2,598.94) = 3.938; *p* = 0.020; *ICC* = 0.057
	Control	39.95 ± 10.99	38.66 ± 10.68	39.09 ± 10.82	−3.23%; *d* = −0.12	−2.15%; *d* = −0.08	
Working memory	Experimental	1048.62 ± 228.20	890.00 ± 158.84	915.00 ± 166.47	−15.13%; *d* = −0.80	−12.74%; *d* = −0.66	*F*(2,598.82) = 6.091; *p* = 0.020; *ICC* = 0.054
	Control	1103.15 ± 233.64	1069.99 ± 197.81	1080.00 ± 201.75	−3.01%; *d* = −0.15	−2.10%; *d* = −0.11	
Cognitive flexibility	Experimental	551.18 ± 149.76	480.00 ± 153.99	502.50 ± 162.33	−12.91%; *d =* −0.47	−2.15%; *d* = −0.08	*F*(2,601.00) = 2.575; *p* = 0.077; *ICC* = 0.000
	Control	547.26 ± 148.53	535.00 ± 78.68	540.00 ± 81.37	−2.24%; *d* = −0.10	−1.33%; *d* = −0.06	

All values are presented as mean ± standard deviation (M ± SD). Percentage changes reflect the rate of improvement from pre-test to post-test or follow-up. Cohen’s *d* values represent effect sizes, where *d* = 0.2 is small, *d* = 0.5 is medium, and *d* ≥ 0.8 is large. Lower values in response time indicate better executive function. ICC = Intraclass correlation coefficient. Linear mixed-effects models were used to account for the clustered study design.

#### 3.2.3 Cognitive flexibility

Linear mixed-effects model analysis showed a time × group interaction effect that approached significance for cognitive flexibility [*F*(2, 601.0) = 2.575, *p* = 0.077, ICC = 0.000], indicating a trend toward differential change patterns between groups across time points (see Figure 6).

Simple effects analysis revealed significant within-group changes for the experimental group: performance improved from baseline to post-test (*t* = 71.182, *p* < 0.001) and from baseline to follow-up (*t* = 48.682, *p* = 0.029), corresponding to a 12.91% reduction in reaction time at post-test (*d* = −0.47) and an 8.83% improvement at follow-up (*d* = −0.32). The control group showed minimal changes across all time points.

Between-group comparisons revealed no significant differences at baseline. The experimental group showed better cognitive flexibility performance than the control group at both post-test and follow-up, though the overall interaction effect approached but did not reach conventional significance levels. Table 2 shows the descriptive statistics and effect sizes for both groups across all time points. Cohen’s *d* for both the CEPA and control groups at pre-test, post-test, and follow-up.

### 3.3 The intervention effect on self-control

Linear mixed-effects model analysis revealed significant time × group interaction effects for the total score of self-control [*F*(2, 599.2) = 66.118, *p* < 0.001, ICC = 0.040], lifestyle habits [*F*(2, 598.3) = 9.790, *p* < 0.001, ICC = 0.006], social interaction [*F*(2, 601.0) = 61.480, *p* < 0.001, ICC = 0.000], emotional experience [*F*(2, 599.4) = 8.792, *p* < 0.001, ICC = 0.004], and learning behavior [*F*(2, 599.1) = 18.715, *p* < 0.001, ICC = 0.072], indicating different changing patterns in self-control performance between the experimental and control groups across time points (see Figure 6). The mean values (M ± SD), variation amplitudes, and effect sizes for each index at different time points are shown in Table 3.

**Table 3:** Descriptive statistics and mixed-effects model results for self-control measures.

Measure	Group	Pre-test	Post-test	Follow-up	Pre-post change	Pre-follow-up change	Mixed-effects model results
Self-control	Experimental	109.15 ± 11.65	137.94 ± 17.00	131.05 ± 8.54	+26.38%; *d* = 1.98	+20.06%; *d* = 2.14	*F*(2, 599.18) = 66.118; *p* < 0.001; *ICC* = 0.040
	Control	108.61 ± 12.04	111.47 ± 11.79	110.24 ± 9.67	+2.63%; *d* = 0.24	+1.50%; *d* = 0.15	
Lifestyle habits	Experimental	22.10 ± 4.20	26.51 ± 7.51	26.19 ± 3.48	+19.91%; *d* = 0.74	+18.51%; *d* = 1.05	*F*(2, 598.29) = 9.790; *p* < 0.001; *ICC* = 0.006
	Control	22.04 ± 4.12	22.69 ± 4.51	22.32 ± 5.14	+2.95%; *d* = 0.15	+1.27%; *d* = 0.06	
Social interaction	Experimental	18.07 ± 3.62	26.80 ± 4.66	21.00 ± 3.20	+48.31%; *d* = 2.06	+16.22%; *d* = 0.85	F(2, 599.43) = 8.792; *p* < 0.001; *ICC* = 0.004
	Control	17.99 ± 3.69	18.12 ± 3.66	18.07 ± 4.79	+0.72%; *d* = 0.04	+0.44%; *d* = 0.02	
Emotional experience	Experimental	9.01 ± 3.33	12.06 ± 3.66	10.51 ± 2.88	+33.85%; *d* = 0.87	+16.65%; *d* = 0.48	*F*(2, 599.43) = 61.480; *p* < 0.001; *ICC* = 0.004
	Control	8.53 ± 3.35	8.75 ± 3.65	8.76 ± 3.58	+2.58%; *d* = 0.06	+2.70%; *d* = 0.07	
Learning behavior	Experimental	59.97 ± 9.94	72.58 ± 13.72	73.34 ± 8.09	+21.03%; *d* = 1.06	+22.29%; *d* = 1.45	*F*(2, 599.10) = 18.715; *p* < 0.001; *ICC* = 0.072
	Control	59.94 ± 10.06	61.81 ± 9.57	60.88 ± 15.16	+3.12%; *d* = 0.19	+1.57%; *d* = 0.07	

All values are presented as mean ± standard deviation (M ± SD). Percentage changes reflect the rate of improvement from pre-test to post-test or follow-up. Cohen’s *d* values represent effect sizes, where *d* = 0.2 is small, *d* = 0.5 is medium, and *d* ≥ 0.8 is large. Higher scores represent better self-control performance. ICC = Intraclass correlation coefficient. Linear mixed-effects models were used to account for the clustered study design.

For the total score of self-control, simple effects analysis showed significant within-group changes for the experimental group, with a significant increase in score after the intervention (*t* = 28.796, *p* < 0.001, *d* = 1.98). Although the follow-up score was lower than the post-test measurement (*t* = 6.893, *p* < 0.001, *d* = 0.46), it remained higher than baseline (*t* = 21.903, *p* < 0.001, *d* = 1.46). The control group showed minimal changes across all time points. Between-group comparisons showed no significant difference at baseline, but the experimental group was significantly better than the control group at both post-test and follow-up.

For lifestyle habits, the experimental group’s post-test score was significantly higher than the baseline score (*t* = 4.408, *p* < 0.001, *d* = 0.74), and this improvement was maintained at follow-up (*t* = 4.097, *p* < 0.001, *d* = 1.05). No significant change was observed in the control group. Between-group comparison showed that the experimental group was significantly superior to the control group at both post-test and follow-up stages.

For social interaction, the experimental group’s score significantly increased after the intervention (*t* = 8.728, *p* < 0.001, *d* = 2.06), and although it decreased during the follow-up stage (*t* = 5.796, *p* < 0.001, *d* = 1.46), it remained higher than baseline (*t* = 2.932, *p* < 0.001, *d* = 0.85). No significant change was observed in the control group. Between-group comparison showed that the experimental group was significantly superior to the control group at both post-test and follow-up stages.

For emotional experience, the experimental group’s post-test score significantly increased (*t* = 3.049, *p* < 0.001, *d* = 0.87), and although it decreased during follow-up (*t* = 1.544, *p* = 0.004, *d* = 0.45), it remained higher than baseline levels (*t* = 1.505, *p* = 0.005, *d* = 0.47). The control group showed minimal changes across all time points. Between-group comparison showed that the experimental group was significantly better than the control group at both post-test and follow-up stages.

For learning behavior, the experimental group’s post-test score was significantly higher than the baseline score (*t* = 12.612, *p* < 0.001, *d* = 1.06), and no significant difference was found between follow-up and post-test scores, but it remained better than baseline (*t* = 13.369, *p* < 0.001, *d* = 1.45). No significant change was observed in the control group. Between-group comparison showed that the experimental group was significantly superior to the control group at both post-test and follow-up stages.

## 4 Discussion

### 4.1 Overview of the main findings

This study implemented a 10-week CEPA intervention, systematically examining its temporal effects on EF and self-control abilities in younger school-aged children. Results indicated that CEPA significantly enhanced EF and self-control in younger school-aged children, and these improvements largely persisted 3-months post-intervention, confirming the primary hypothesis.

The primary findings are summarized into three aspects: First, Hypotheses 1 and 3 were supported. CEPA significantly improved the three core EF components (inhibitory control, working memory, and cognitive flexibility), with these improvements largely sustained at follow-up. Second, Hypothesis 2 was partially supported. The three EF components exhibited distinct improvement patterns: working memory demonstrated the greatest immediate effect, inhibitory control showed the highest persistence, while cognitive flexibility improved relatively less. Finally, Hypothesis 4 was supported. CEPA effectively enhanced children’s self-control, particularly in social interaction and learning behavior. In contrast, the Traditional Physical Education Curriculum showed no significant changes across all measured indicators.

Overall, the intervention effects followed a temporal pattern of “rapid improvement, slight attenuation, and stable maintenance,” consistent with the assumption of a nonlinear temporal process. These findings confirm CEPA effectiveness and provide critical evidence regarding the temporal dynamics of intervention outcomes. These results offer valuable empirical evidence for future theoretical studies and practical applications, particularly in educational contexts and behavioral interventions for children.

### 4.2 Effects and mechanisms of CEPA on executive function in younger school-aged children

This study demonstrated that CEPA significantly improved EF in younger school-aged children, especially in inhibitory control, working memory, and cognitive flexibility. At post-test, inhibitory control, working memory, and cognitive flexibility increased by 15.47% (*d* = − 0.66), 15.13% (*d* = −0.80), and 12.91% (*d* = −0.47), respectively, in the experimental group. These improvements maintained moderate to large effect sizes at the three-month follow-up (inhibitory control *d* = −0.58, working memory *d* = −0.66), suggesting persistent EF enhancement from CEPA.

Notably, improvements differed significantly among the three core EF components, reflecting distinct component-specific and nonlinear temporal characteristics. Working memory exhibited the strongest immediate effects, while inhibitory control showed the greatest persistence. In contrast, cognitive flexibility improved to a lesser extent and demonstrated greater decline during the follow-up period. This nonlinear temporal characteristic aligns with Pesce’s cognitive motor adaptation theory (Pesce et al., 2016), wherein EF improves rapidly initially before stabilizing.

Two core theoretical mechanisms may explain the observed EF improvements. First, neural plasticity theory suggests CEPA enhances efficiency in EF-related brain regions by increasing brain-derived neurotrophic factor secretion, thus promoting neural plasticity and prefrontal cortex connectivity (Hillman et al., 2008). Research by Ludyga confirmed significantly reduced oxygenation differences in the prefrontal cortex of children during inhibitory tasks following acute cognitive-motor interventions, indicating improved neural efficiency (Ludyga et al., 2024). This finding aligns with previous studies utilizing functional near-infrared spectroscopy and electroencephalogram (EEG), which demonstrated that motor interventions regulate brain activity, thereby facilitating the development of working memory and inhibitory control, providing additional neural-level evidence (Mora-Gonzalez et al., 2024; Wang et al., 2023). Second, the cognitive engagement hypothesis proposes that cognitive challenges in physical activities effectively activate shared neural circuits for motor and cognitive functions, enhancing activity and integration in EF-related brain regions, particularly the prefrontal cortex (Best, 2010). The CEPA design in this study incorporated a gradient cognitive load, progressively increasing task complexity and cognitive challenges to optimize efficiency in shared neural networks. Consequently, significant and lasting improvements occurred in EF components with high attentional demands, such as working memory and inhibitory control.

These persistent improvements in inhibitory control are consistent with findings from Egger et al. (2019) and Schmidt et al. (2015). These researchers similarly observed persistent effects of cognitive-motor intervention on inhibitory control. Continuous impulse control training within sports contexts may be particularly effective for enhancing this EF component. The notable improvement in working memory aligns with findings by Pesce, likely due to cognitive-motor tasks requiring substantial information retention and manipulation, thus offering extensive training opportunities for working memory (Pesce, 2012).

However, the relatively limited and less persistent improvement in cognitive flexibility requires further investigation. Research by Anzeneder further suggests that cognitive challenge level significantly influences intervention effectiveness. Cognitive loads that are excessively high or low may reduce intervention effectiveness (Anzeneder et al., 2023). Cognitive flexibility requires frequent cognitive task-switching and rapid strategic adjustments, potentially exceeding optimal cognitive challenge ranges. Thus, improvement effects may be limited. Furthermore, previous studies indicated distinct developmental trajectories and plasticity windows for different EF components (Bellaj et al., 2016). Inhibitory control develops rapidly during younger school-aged and is relatively sensitive to interventions. Cognitive flexibility, as a more advanced EF component, may require a longer intervention period for significant improvement (Korzeniowski et al., 2021).

In conclusion, this study confirmed the effectiveness of CEPA in enhancing EF, revealing distinct improvement characteristics and neural mechanisms for each EF component. These findings offer critical theoretical and empirical insights into the mechanisms by which CEPA promotes EF development and provide practical guidance for future intervention research.

### 4.3 Effects and mechanisms of CEPA on self-control in younger school-aged children

The results indicated that CEPA significantly enhanced self-control in younger school-aged children. The total self-control score in the CEPA group increased by 26.38% (*d* = 1.98) at post-test and remained significantly elevated by 20.06% (*d* = 2.14) at follow-up. In contrast, the control group exhibited minimal changes of 2.63% (*d* = 0.24). CEPA significantly improved all self-control sub-dimensions, though the extent of improvement varied considerably: Social interaction improved most significantly (48.31%, *d* = 2.06), followed by emotional experience (33.85%, *d* = 0.87), learning behavior (21.03%, *d* = 1.06), and lifestyle habits (19.91%, *d* = 0.74). In contrast, changes across all dimensions were minimal (3.12%) in the Traditional Physical Education Curriculum, with relatively small effect sizes (*d* = 0.04–0.19). These results highlight the significant and substantial effects of CEPA on self-control enhancement. Self-control improvements exhibited two main characteristics. First, intervention effects differed across sub-dimensions, with social interaction and emotional experience improving more substantially than learning behavior and lifestyle habits. Second, the improvements were sustained, remaining evident at the follow-up stage.

The mechanisms underlying self-control improvement may be explained from two perspectives. First, from a neurobiological perspective, CEPA may enhance emotion and behavior regulation by promoting functional development in key self-regulatory brain regions (Yoshimura et al., 2014), such as the prefrontal cortex and anterior cingulate cortex (van Noordt and Segalowitz, 2012). Research by Chao et al. indicated that exercise interventions enhance functional connectivity between the prefrontal cortex and limbic system, crucial for emotional regulation and social behavior control (Chao et al., 2021). Second, from a psychological perspective, CEPA provides numerous situations requiring self-control, such as rule compliance, strategic adjustments, turn-taking, and cooperative interactions. These experiences offer practical opportunities for self-control, facilitating the development of relevant skills (Gülseven et al., 2021). According to Baumeister’s “Self-control Strength Model” (Baumeister et al., 2007), self-control can be strengthened through repeated practice, analogous to muscle strengthening through exercise. The diverse challenges offered by CEPA may effectively train children’s self-control skills, particularly through repeated practice in social behavior regulation, emotional management, and learning behavior adjustments, thus enhancing efficiency in related neural pathways.

Improvements in social interaction observed in this study align with findings by Vazou and Zhao, who reported that structured physical activities effectively enhance children’s social behavior regulation skills (Vazou et al., 2017; Zhao and Chen, 2018). This may be associated with the abundant social interaction elements integrated into CEPA. The intervention incorporated numerous elements, including teamwork, rule compliance, and interpersonal coordination, providing frequent opportunities to practice social behavior regulation and thus enhancing children’s social interaction skills (Harrell et al., 2009). The improvement in emotional experience aligns with findings from Flook et al. regarding mind–body interventions (Flook et al., 2010). Although CEPA does not directly target emotion regulation training, it may indirectly foster children’s emotional regulation by offering opportunities to overcome challenges, experience success, and manage failure (Cole et al., 2009). Although learning behaviors and lifestyle habits improved significantly, the magnitude was relatively smaller, consistent with Wood’s conclusion that changing habitual behaviors requires more sustained intervention and practice (Wood and Neal, 2016).

Notably, the substantial improvement in social interaction (48.31%) exceeded effect sizes typically reported in previous research. Pandey et al. meta-analysis reported average effect sizes of approximately *d* = 0.3–0.5 for interventions targeting children’s social skills (Pandey et al., 2018). The notably larger improvement in this study may reflect CEPA’s unique advantage of combining cognitive challenges with social interaction, producing a synergistic effect. Bridgett et al. proposed a close relationship between cognitive control and social behavior (Bridgett et al., 2013), which the findings of this study further support.

The findings of this study hold significant theoretical and practical implications. From a theoretical perspective, the results support the multi-dimensional structural model of self-control and reveal that sub-dimensions exhibit differential sensitivities to interventions. This finding enriches the understanding of self-control developmental mechanisms, suggesting distinct developmental trajectories and intervention windows across sub-dimensions. Practically, considering self-control’s importance for children’s academic performance, social adaptation, and mental health (Moffitt et al., 2011), CEPA represents a valuable educational strategy to foster self-control development. Schools and educators may consider integrating CEPA into regular curricula, particularly to enhance social interaction and emotional regulation skills. Future research may explore strategies for effectively targeting learning behaviors and lifestyle habits to promote comprehensive self-control development.

### 4.4 Temporal characteristics of CEPA intervention effects in younger school-aged children

This study utilized data collected at three time points (pre-test, post-test, and three-month follow-up) to reveal the temporal characteristics of CEPA intervention effects. Results indicated a distinct nonlinear temporal pattern of intervention effects, with significantly greater improvements during the intervention period (0–10 weeks) compared to the maintenance period (10 weeks–3 months). Executive function components showed significant improvements during the intervention period (0–10 weeks), with inhibitory control showing a 15.47% improvement (*d* = −0.66), working memory demonstrating a 15.13% improvement (*d* = −0.80), and cognitive flexibility achieving a 12.91% improvement (*d* = −0.47). These gains were largely maintained during the follow-up period (10 weeks–3 months). Total self-control scores increased by 26.38% (*d* = 1.98) during the intervention and declined by 5.15% (*d* = −0.31) during maintenance. This pattern suggests that despite a slight decline post-intervention, intervention effects remained significantly higher than baseline overall.

CEPA intervention effects exhibited three main characteristics: immediacy, differential impact, and nonlinear progression. First, regarding immediacy, CEPA positively impacted EF and self-control within a relatively short duration (10 weeks). This immediate effect may result from the high engagement and challenge level of CEPA, prompting rapid mobilization and practice of relevant cognitive resources (Best, 2010). Compared to the traditional physical education curriculum, CEPA organically integrates cognitive challenges with physical activities, activating broader neural networks and accelerating the onset of intervention effects.

Second, the temporal progression of intervention effects exhibited cross-domain differences. Overall, self-control showed greater immediate improvements compared to EF but also demonstrated relatively greater attenuation during the maintenance period. This difference reflects a hierarchical “inside-out” model of cognitive-behavioral changes (Zelazo and Carlson, 2012). EF, as a fundamental cognitive ability, may induce more stable neural network changes, whereas self-control, as a phenotypic behavior, is influenced by environmental factors alongside basic neural modifications. Research by Tomporowski et al. supports this perspective. They found that cognitive improvements typically precede and stabilize changes in behavioral performance (Tomporowski et al., 2015a; Tomporowski et al., 2015b). Finally, the nonlinear temporal pattern of intervention effects may reflect the biological processes underlying neural connection formation and consolidation. The “neural adaptation window” theory by Davis et al. proposes that neural plasticity changes induced by cognitive training follow a specific temporal trajectory: an initial rapid formation phase, a mid-term consolidation phase, and a long-term maintenance phase (Davis et al., 2011). The observed pattern of rapid improvement during intervention followed by a relatively stable maintenance period aligns closely with this theoretical framework.

The temporal characteristics of intervention effects provide theoretical support for understanding the long-term efficacy of cognitive interventions. Theoretically, results support nonlinear models of cognitive development and phased neural plasticity changes (Molenaar and Raijmakers, 2000), with the pattern of “rapid improvement–slight attenuation–stable maintenance” highlighting potential critical windows for cognitive development. Practically, the persistence of intervention effects over 3 months indicates children can largely maintain cognitive and behavioral improvements even after intervention cessation. These findings offer empirical support for implementing CEPA interventions within school settings. Future research could investigate longer-term maintenance patterns (e.g., 6 months or 1 year) and develop optimal enhanced intervention programs to sustain and further enhance children’s cognitive and behavioral development.

### 4.5 Practical implications

The findings have broad practical implications, particularly for educational practices and child developmental interventions. First, as an innovative educational approach, CEPA provides new perspectives for reforming school physical education curricula. The traditional physical education curriculum usually focuses solely on physical fitness improvements, neglecting the development of cognitive and behavioral regulation skills. This study confirmed that integrating cognitive challenges into physical activities simultaneously enhances children’s EF and self-control skills. Thus, school physical education curricula should transition from solely physical training toward a mind–body integrated development model (Diamond, 2015). Specifically, schools can foster comprehensive cognitive and behavioral development by reforming traditional curricula and incorporating cognitive challenge elements such as teamwork, strategic planning, and rule modifications.

Second, the design principles and implementation methods of CEPA offer practical operational guidance for educators and intervention specialists. The principle of gradient cognitive load suggests gradually adjusting intervention difficulty based on children’s ability levels to maintain optimal challenges. This principle provides critical guidance for teachers in task design, preventing tasks from being overly simple and ineffective or excessively difficult and frustrating (Pesce et al., 2019).

Third, CEPA has potential clinical application value for children with special developmental needs. Previous studies indicated that deficits in EF and self-control are core symptoms of various childhood developmental disorders, including Attention Deficit Hyperactivity Disorder (ADHD), learning disabilities, and Autism Spectrum Disorders (ASD) (Corbett et al., 2009). This study confirmed that CEPA significantly improved EF and self-control skills in younger school-aged children, suggesting potential therapeutic value for children exhibiting EF deficits. Compared to traditional cognitive training methods, CEPA is characterized by high engagement and interest, potentially enhancing compliance and intervention outcomes for children with special needs. Finally, implementing CEPA does not require expensive equipment or specialized venues. It can be easily promoted and implemented in regular school and community settings, demonstrating high feasibility and cost-effectiveness. The intervention plan used in this study can be flexibly adapted to various contexts, including classroom teaching, extracurricular activities, and family interventions.

In conclusion, this study’s findings hold both significant theoretical implications and substantial practical value. As an innovative educational approach for developmental promotion, CEPA effectively enhances younger school-aged children’s EF and self-control, offering new possibilities for child development interventions. Educators, clinicians, and policymakers can utilize these findings to develop and implement more effective child developmental programs, enhancing children’s cognitive and behavioral outcomes.

### 4.6 Research limitations and prospects

Although this study yielded positive findings, several limitations remain. First, the absence of data from additional time points (e.g., monthly or weekly) limited the ability to evaluate dynamic changes in intervention effects. Second, although behavioral tasks and self-report measures provided valuable insights, future studies could integrate physiological indicators (e.g., EEG, HRV) for a more comprehensive assessment of CEPA intervention effects. Additionally, the study samples were primarily from schools in a single region, consisting mostly of younger school-aged children from general educational backgrounds. Thus, the generalizability of results may be limited by regional and cultural factors.

Future research should expand sample diversity to include broader child populations. Future research directions include: (1) extending the intervention duration, increasing follow-up periods, and utilizing more frequent assessments (e.g., monthly) to examine persistence and temporal patterns of intervention effects. (2) Adopting multidimensional assessment methods by integrating physiological measurements (e.g., EEG, HRV) with behavioral data for comprehensive evaluations. This approach would more objectively reveal the specific effects of CEPA on children’s EF and self-control skills. (3) Enhancing generalizability by expanding sample sizes to include children from diverse regional, cultural, and socioeconomic backgrounds. Additionally, future studies could investigate the effects of CEPA on special populations (e.g., children with ADHD, ASD) to verify intervention applicability and effectiveness.

## 5 Conclusion

This study systematically evaluated the effects of CEPA on EF and self-control in younger school-aged children and examined its temporal characteristics. The main findings can be summarized into three points: First, CEPA effectively enhanced three core EF components (inhibitory control, working memory, and cognitive flexibility), with inhibitory control showing the greatest persistence. Second, the intervention significantly improved four self-control dimensions (social interaction, emotional experience, learning behavior, and lifestyle habits), with social interaction improvements being particularly prominent. Third, the intervention effects exhibited a clear nonlinear temporal pattern, indicating that cognitive and behavioral developments follow specific temporal trajectories.

Theoretically, this study supports the “Cognitive Engagement Hypothesis” and “Cognitive Adaptation Threshold Theory,” verifying the distinct advantages of integrating cognitive challenges into physical activities. Practically, these findings provide novel insights for reforming school physical education curricula and designing child developmental interventions. Educators are advised to integrate cognitive challenges into physical activities and regularly implement CEPA to sustain long-term benefits. Despite limitations in sample representativeness and study design, these findings offer critical evidence on the plasticity of cognitive and behavioral development in younger school-aged children, providing a scientific foundation for future educational and developmental intervention programs.

## Data Availability

The raw data supporting the conclusions of this article will be made available by the authors, without undue reservation.

## Footnotes

^1^ https://www.chictr.org.cn/showproj.html?proj=209066

## Supplementary material

SUPPLEMENTARY TABLE 1 10-week intervention course plan.
